# In Depth Natural Product Discovery from the Basidiomycetes *Stereum* Species

**DOI:** 10.3390/microorganisms8071049

**Published:** 2020-07-15

**Authors:** Mengqing Tian, Peiji Zhao, Guohong Li, Keqin Zhang

**Affiliations:** State Key Laboratory for Conservation and Utilization of Bio-Resources in Yunnan, and Key Laboratory for Southwest Microbial Diversity of the Ministry of Education, Yunnan University, Kunming 650032, China; 15198961953@163.com (M.T.); pjzhao@ynu.edu.cn (P.Z.)

**Keywords:** Basidiomycetes, *Stereum*, metabolites, bioactivity

## Abstract

Natural metabolites from microorganisms play significant roles in the discovery of drugs, both for disease treatments in humans, and applications in agriculture. The Basidiomycetes *Stereum* genus has been a source of such bioactive compounds. Here we report on the structures and activities of secondary metabolites from *Stereum*. Their structural types include sesquiterpenoids, polyketides, vibralactones, triterpenoids, sterols, carboxylic acids and saccharides. Most of them showed biological activities including cytotoxic, antibacterial, antifungal, antiviral, radical scavenging activity, autophagy inducing activity, inhibiting pancreatic lipase against malarial parasite, nematocidal and so on. The syntheses of some metabolites have been studied. In this review, 238 secondary metabolites from 10 known species and various unidentified species of *Stereum* were summarized over the last seven decades.

## 1. Introduction

Natural products deriving from microorganisms are one of the most valuable sources for medicinal and agricultural applications [1,2,3]. Basidiomycetes, as producers of bioactive secondary metabolites, provide an exciting opportunity to obtain new natural products with high application potential [4]. The increasing number of new and active compounds recently discovered in Basidiomycetes have demonstrated their capacity for producing many more unknown natural products, most of which are still unexploited for their applications.

*Stereum* belongs to the family of Stereaceae (Basidiomycetes), which is mainly distributed in tropical and subtropical areas, and it often lives in woody debris, rotting trunks and sometimes on buried dead wood [5]. There are 27 species in *Stereum*, according to the Dictionary of Fungi (http://www.speciesfungorum.org/Names/Fundic.asp, January, 2020). *Stereum* spp. are known to be producers of biologically active secondary metabolites. Previous investigations recorded a number of interesting new compounds isolated from *Stereum*, especially sesquiterpenoids, and about ten types of sesquiterpenoids were obtained from the genus, so it is one of the major sources of structurally diverse sesquiterpenes. Secondary metabolites from *Stereum* had diversiform biological activities, such as cytotoxic, antimicrobial, inhibit pancreatic lipase, nematocidal and so on. Some of them showed promising activities, for example, vibralactone, isolated from *Stereum vibrans*, is a lead compound with a novel skeleton structure and strong inhibitory activity (IC_50_ 0.4 μg/mL) against pancreatic lipase, which is a potential candidate for a new antiobesity therapeutic [6,7]. Up to now, there are 10 known species and various unidentified species of *Stereum* have been investigated for their metabolites. This review presents 238 secondary metabolites isolated from the *Stereum* spp., including their structures and biological activities reported over the last seven decades.

## 2. Secondary Metabolites from *Stereum* and Their Activities

### 2.1. Sesquiterpenoids

Sesquiterpenoids have various structures and abundant bioactivities. Sesquiterpenoids from *Stereum* include hirsutane, illudalane, norilludalane, sterpurane, cadinane, drimane, isolactarane, stereumane and other types. 

#### 2.1.1. Hirsutane

**Figure 1:**
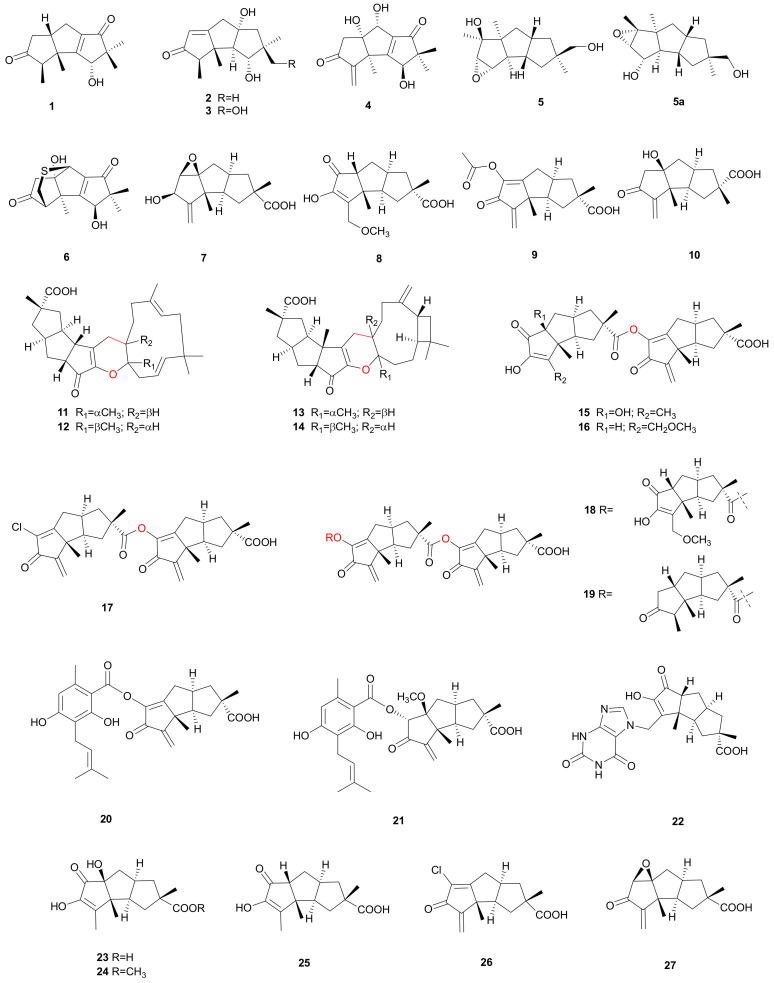
Hirsutane sesquiterpenoids from *Stereum*.

Most of the hirsutane sesquiterpenoids have been isolated from *Stereum hirsutum*, which can cause disease in grapevine and also seems to be related to wood deterioration [8]. The hirsutane sesquiterpenes (Figure 1) obtained from *S. hirsutum*, includes hirsutenols A–F (1–6) [9,10], hirsutic acids C-E, N (7–10) [11,12], sterhirsutins A–L (11–22) [12,13], and sesquiterpenoids 23–25 [14]. Hirsutic acid C (7) was assigned by a combination of chemical and X-ray studies [15]. The absolute configurations of sterhirsutins A (11) and B (12) (assigned as 2R, 3S, 7R, 9S, 11S, 16S, 17R and 2R, 3S, 7R, 9S, 11S, 16R, 17S) were confirmed by X-ray experiments and electronic circular dichroism (ECD) calculations [12]. The absolute configuration of sterhirsutin C (13) was assigned as 2R, 3S, 7R, 9S, 11S, 16S, 17R, 20S, and 23R by an X-ray experiment. Hirsutenols A–C (1–3) showed moderate activity against *Escherichia coli* by the agar diffusion method (50 μg/disk) [9]. Hirsutenols D–F (4–6) presented strong scavenging activity against superoxide anion radicals and the EC_50_ values were 8.78, 1.62 and 0.39 mM, respectively [10]. Free radical scavengers are regarded as protective agents against related disorders, such as atherosclerosis, myocardial and cerebral ischemia, diabetes, cancers, rheumatoid arthritis and aging processes [16,17,18]. In 2017, Kutateladze et al. validated and corrected the structure of hirsutenol E (5a) using the density functional theory (DFT) computational method [19]. Hirsutic acid C (7) is a precursor to an antibacterial substance, hirsutic acid N (10) [11]. Hirsutic acids C (7) and D (8) showed cytotoxicity against K562 cells with IC_50_ values of 6.93 and 30.52 μg mL^-1^, respectively; they also showed cytotoxicity to the HCT116 cell line with IC_50_ values of 25.43 and 24.17 μg mL^-1^, respectively [12]. Sterhirsutins A (11) and B (12) are likely biosynthesized by a hetero-Diels-Alder cycloaddition from a hirsutane type sesquiterpenoid and α-humulene. Sterhirsutins A–L (11–22) showed weak cytotoxicity against HCT116 and K562 cell lines [12]. The autophagy inducing activities are related to the therapy of cancer and neurodegenerative disorders. The quantification of GFP-LC3 expression in cells is an indicator to evaluate the autophagy inducing effect. Taking advantage of this method, sterhirsutins J (20) and K (21) exhibited strong autophagy inducing activity at a concentration of 50 μM. Sterhirsutin G (17) may be a potential drug in the therapy of autoimmune diseases because of its ability to inhibit the RLRs-mediated antiviral signaling in cells. The ether bond between C-15’ and C-16’ may be significant for immunosuppressant bioactivity because that sterhirsutin E (15) showed no inhibitory activity on the IFNβ promoter activation in comparison with sterhirsutin G (17) [13]. Sesquiterpenoids 23–25 showed weak radical scavenging activities with EC_50_ values larger than 200 μM. Sesquiterpenoid 23 displayed strong NO inhibitory activity in lipopolysaccharide-induced macrophages with an IC_50_ value of 15.44 ± 1.07 μM and showed strong cytotoxicity towards HepG2 with an IC_50_ value of 24.41 ± 1.86 μM [16].

Apart from *S. hirsutum*, hirsutane sesquiterpenoids also exist in other strains. Chlorostereone (26) and complicatic acid (27) have been isolated from strain IBWF01082 of *Stereum*, and they probably share the same biosynthetic pathway [20]. Chlorostereone (26) showed cytotoxicity against Jurkat cells with an IC_50_ value of 5 μg mL^−1^. Complicatic acid (27) was first obtained in 1973 from *Stereum complicatum*, and it showed broad but moderate biological activities against numbers of microorganisms including bacteria and certain fungi [21]. Both hirsutic acid C (7) and complicatic acid (27) have been the subjects of many synthetic studies [22]. The racemic complicatic acid (27) was first synthesized in 1974, which demonstrated that it could be reduced with NaBH_4_ in ethanol to (±)-hirsutic acid [23,24]. In 1985, Schuda et al. reported that hirsutic acid C (7) and complicatic acid (27) could be synthesized from the methanoindene in a highly stereoselective manner [25]. In 2006, Kerrie et al. reported on the exploitation of cis-1,2-dihydrocatechol in the development of total syntheses of hirsutic acid C (7) and complicatic acid (27) [26,27]. In addition to chemical syntheses, scientists are also working on biosynthetic approaches. The biosynthesis of hirsutic acid C (7) and complicatic acid (27) was carried out using ^13^C-stable isotope feeding [24]. Recently, Flynn et al. described the cloning and functional characterization of a hirsutene synthase, which combined a sesquiterpene synthase (STS) with a C-terminal 3-hydroxy-3-methylglutaryl-coenzyme A (3-hydroxy-3-methylglutaryl-CoA) synthase (HMGS) domain [28]. Their structures are shown in Figure 1.

#### 2.1.2. Illudalane and Norilludalane

Three novel dimeric sesquiterpenoids, sterostreins A–C (28–30), and twelve illudalanes and norilludalanes, sterostreins D–O (31–42), were obtained from cultures of *S. ostrea* BCC 22955 [29,30]. Sterostreins D–H (31–35) are novel furan-containing tricyclic illudalanes; sterostreins J–L (37–39) are new norilludalanes, and M–O (40–42) are pyridine-containing tricyclic illudalanes. The absolute configuration of sterostrein D (31) was confirmed by its 8β-hydroxy derivative using the modified Mosher’s method [29]. Sterostrein A (28) and sterostrein C (30) were also isolated from *Stereum* sp. YMF1.1686 [31], and sterostrein H (35) from *Stereum* sp. NN048997 [32] and *S. hirsutum* FP-91666 [33]. Sterostrein A (28) displayed cytotoxicity against the cancer cell lines MCF-7, KB, and NCI-H187 with IC_50_ values of 7.2, 38, and 5.3 μg mL^−1^, respectively. This compound also exhibited activity against the malarial parasite *Plasmodium falciparum* K1 with an IC_50_ of 2.3 μg mL^−1^.

**Figure 2:**
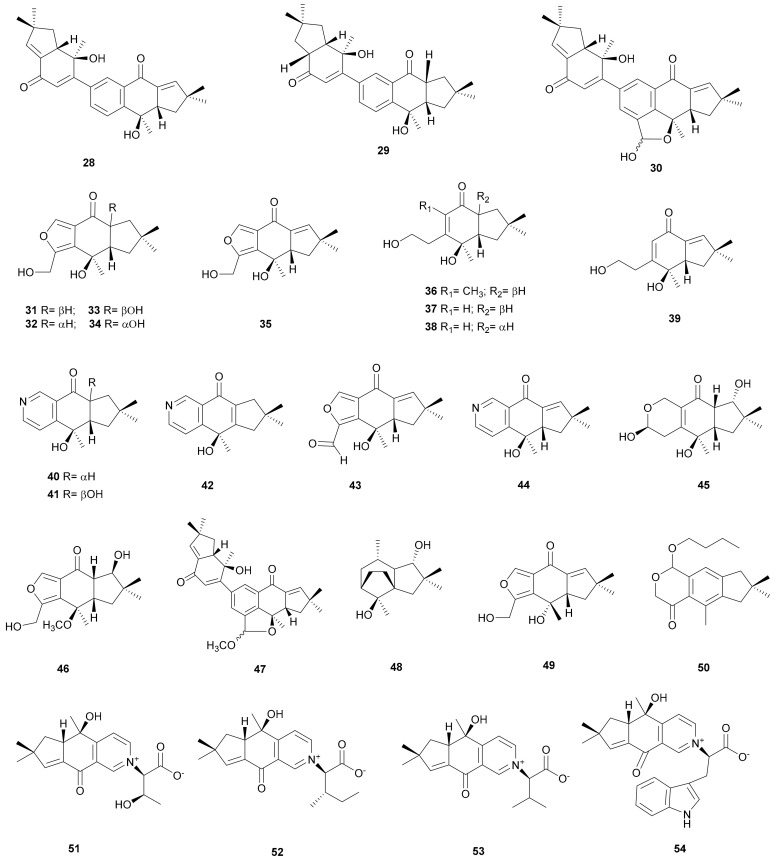
Illudalane and norilludalane sesquiterpenoids from *Stereum*.

Sterostrein D (31) showed weak cytotoxicity towards KB cells (IC_50_ 26 μg mL^-1^) [30]. Sterostreins P (43) and Q (44) were isolated from the culture broth of *Stereum* sp. NN048997. Sterostrein P (43) showed weak nematocidal activity against the *Caenorhabditis elegans* [32]. Sterostrein Q (44) was also isolated from the YMG broth of *S. hirsutum* FP-91666 and showed antibacterial activity against *Salmonella typhimurium*, *Staphylococcus aureus*, and *E. coli*, with minimum inhibitory concentration (MIC) values of 50.0, 25.0 and 12.5 μg mL^−1^, respectively [33]. Sterostreins R-U (45-48) were isolated from *Stereum* sp. YMF1.1686 [31,34]. Sterostreins V (49) and W (50) were obtained from *Stereum* sp. YMF1.04734 and *S. rugosum* ATCC64657, respectively [35,36]. Four terpenes, stereumamides A–D (51–54) were isolated from the YMG broth of *S. hirsutum* FP-91666. The author believed that they are the first example of a naturally occurring quaternary ammonium compound (QAC) of sesquiterpenes combining with α-amino acids. Stereumamides A (51) and D (54) showed antibacterial activity against *Salmonella typhimurium*, *Staphylococcus aureus,* and *E. coli*, with MIC values between 12.5-25.0 μg mL^−1^ [33]. Their structures are shown in Figure 2.

#### 2.1.3. Stereumane and Cadinane

Ten new sesquiterpenoids, stereumins A–J (55–64), containing a new stereumane-type backbone, were obtained from fungus *Stereum* sp. CCTCC AF 207024 [37,38,39]. The relative configurations of stereumins A (55) and B (56) were further confirmed by X-ray analysis [38].

**Figure 3:**
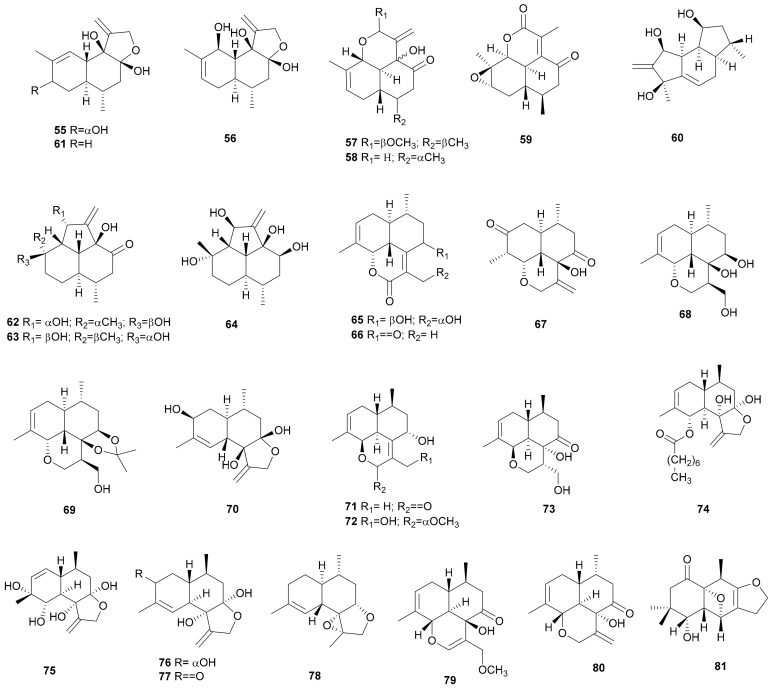
Stereumane and cadinane sesquiterpenoids from *Stereum*.

The absolute configuration of stereumin H (62) was established by X-ray analysis and density functional theory (DFT) calculations. Stereumin I (63) is a diastereoisomer of stereumin H (62), and its absolute configuration was assigned as (1R,2S,3R,6R,7R,8S,12R) using DFT calculations [37]. Stereumins A–E (55–59) showed weak nematocidal activity against the nematode *Panagrellus redivivus* at 400 mg mL^−1^. Among these five compounds, stereumins C (57) and D (58) killed 84.4% and 94.9% of *P. redivivus*, respectively, in 48 h [38]. Stereumins K–P (65–70), namely six new cadinene types, were isolated from *Stereum* sp. CCTCC AF 2012007 [40]. Stereumin O (69) may be formed from stereumin N (68) during isolation. The absolute configuration of stereumin K was established by Cu Kα X-ray analysis [40]. Stereumins Q–U (71–75), together with *ent*-strobilols E (76) and G (77), were isolated from the culture of *S.* cf. *sanguinolentum* BCC 22926 [41]. The absolute configurations of stereumin Q (71) and ent-strobilol E (76) were determined by the modified Mosher’s method. Stereumin T (74) showed antibacterial activity against *Bacillus cereus* with a MIC value of 3.97 μM and cytotoxicity against Vero cell line with an IC_50_ value of 43.7 μM. In addition, stereumins A (55), B (56), K (65), L (66), and N (68) were also isolated from this strain [41]. Strobilols N-P (78-80) were obtained from *S. gausapatum* ATCC60954, among which, strobilol N (78) presented weak nematocidal activity towards *C. elegans* at a concentration of 200 μg mL^−1^ with a fatality rate of 75.8% in 36 h [42]. Another sesquiterpenoid-like structure, determined to be stereumone A (81), was obtained from *Stereum* sp. 8954 [43]. Their structures are shown in Figure 3.

#### 2.1.4. Sterpurane and Isolactarane

The sterpurane is a unique type of sesquiterpenoids possessing an unusual 4/6/5 ring system. These types of compounds, including sterepolide (82), dihydrosterepolide (83), 4,12-dihydroxysterpurene (84), and 5,12-dihydroxysterpurene (85) have been isolated from the fungus *S. purpureum* [44,45]. The synthesis method and biosynthetic process of these metabolites and related compounds have been well studied. Willia et al. found that sterpurenes are formed from acetate through humulene and a protoilludane cation [46]. In 1990, Zhao et al. synthesized the tricyclic sterpurene by a brief route utilizing a cyclopentane annulation as the key transformation [47]. Richard et al. demonstrated that (+)-sterpurene could be synthesized by the concise, enantioselective route using a vinylallene intramolecular Diels-Alder reaction [48]. Goverdhan et al. found a novel synthetic route to sterpurene from cis-diquinane ketone. Their approach used a strategy in which α,β-unsaturated enone moiety of an electron-withdrawing β-substituent promotes an intermolecular [2+2]-photocycloadditionm, which may provide further applications in the synthesis of analogs [22]. Harmata synthesized (±)-sterpurene using a [4+3] cycloaddition approach including a (4 + 3) cycloaddition reaction and a quasi-Favorskii rearrangement [49]. The chemoenzymatic total synthesis of 4,12-dihydroxysterpurene (84) and its enantiomer has also been reported [50].

**Figure 4:**
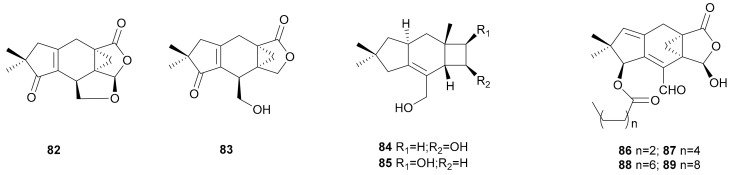
Sterpurane and isolactarane sesquiterpenoids from *Stereum*.

Four tetracyclic sesquiterpenoids possessing an isolactarane skeleton, sterelactones A–D (86–89), have been isolated from *Stereum* sp. IBWF01060 [51]. The four sterelactones showed pronounced antifungal activities, and their IC_100_ values ranged from 1–50 μg mL^−1^ against seven fungi. They also exhibited significant antibacterial and cytotoxic activities. The cytotoxic activity of the four sterelactones was moderate (the IC_50_ values ranged from 10-20 µM), and sterelactone D (89) exhibited weak nematocidal activity towards *C. elegans*, at 100 μg mL^−1^ with a fatality rate of 50% [51]. Their structures are shown in Figure 4.

#### 2.1.5. Drimane

**Figure 5:**
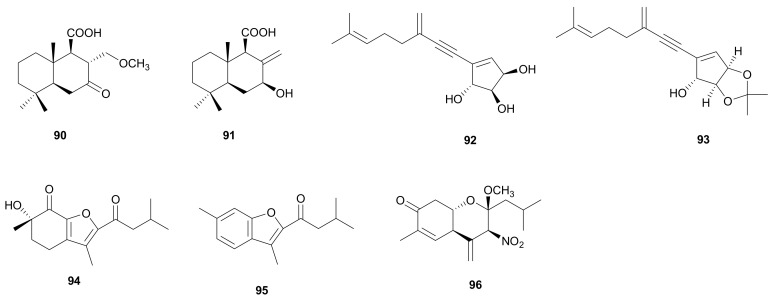
Drimane, acetylenic and unclassified sesquiterpenoids from *Stereum*.

Methoxylaricinolic acid (90) and laricinolic acid (91) (Figure 5), two sesquiterpenes with a drimane skeleton were isolated from the fruit bodies of *S. ostrea* [52]. The two compounds exhibited marginal inhibition against lipid peroxidation in rat liver microsomes with the same IC_50_ values of 50 μg mL^−1^.

#### 2.1.6. Acetylenic Sesquiterpenoids

Stereyne A (92), a novel acetylenic sesquiterpenoid, and its acetonide derivative, stereyne B (93) (Figure 5), which represent a new type of sesquiterpenoids structure, were isolated from the basidiomycete *S.* cf. *hirsutum* BCC 26597 [53]. The absolute configurations of hydroxys were confirmed by modified Mosher’s method.

#### 2.1.7. Unclassified Sesquiterpenoids

Stereumenes A-C (94-96) (Figure 5), three novel sesquiterpenoids, were obtained from the culture broth of *Stereum* sp. YMF1.04183. Stereumene B (95) showed weak nematocidal activity against *C. elegans* with a fatality rate of 41.1% at 200 μg mL^−1^ in 24 h [54]. Showkat et al. found that stereumene B (95) could be synthesized via a TiCl_4_-mediated Tanabe protocol by employing a series of functionalized benzo- and naphthofurans from cyclohexanone precursors [55].

### 2.2. Polyketides and Their Derivatives

Polyketides are a large group of metabolites that have notable variety in their structure and function [56]. The phenolic moiety, orsellinic acid (97), may be derived from acetate units by a polyketide synthase (PKS) [57] and then further modified by reduction, oxidation, and methylation steps. Three new benzoate derivatives 4′-hydroxy-5′-methoxy-6′-(3″-methyl-2″-butenyl)-phenyl- 2,4-dihydroxy-6-methyl-benzoate (98), 4′-hydroxy-6′-(3″-methyl-2″-butenyl)-phenyl-2,4-dihydroxy- 6-methyl-benzoate (99), and MS-3 (100) were isolated from the mycelia of *S. hirsutum* [14,58,59]. Compounds (98) and (99) showed strong inhibitory activity against the growth of methicillin-resistant *Staphylococcus aureus* and *S. aureus* with the same MIC values of 25.0 μg mL^−1^. The two compounds also presented an antibacterial activity against *B. subtilis* with the MIC values of 25.0 and 50.0 μg mL^−1^, respectively and showed radicals scavenging activity with EC_50_ values more than 200 μM. Compound 98 exhibited strong NO inhibitory activity in LPS-induced macrophages with an IC_50_ value of 19.17 ± 1.11 μg mL^−1^ and showed cytotoxicity towards two cell lines, HepG2 and A549. MS-3 (100) had low toxicity on Yoshida rat sarcoma cells in vitro [59], and it was also isolated from the fungus *S. rameale* (N° 2511). MS-3 (100) displayed antibacterial activity against gram-positive bacteria including *B. cereus*, *B. subtilis* and *S. aureus* with MIC values of 50, 10 and 100 μg mL^−1^, respectively [60]. Butyl 2,4-dihydroxy-6-methylbenzoate (101), and orsellinic acid methyl ester (102) were obtained from *Stereum* sp. 8954, and butyl 2,4-dihydroxy-6-methylbenzoate (101) showed significant nematocidal activity against the nematode *P. redivivus* [61].

**Figure 6:**
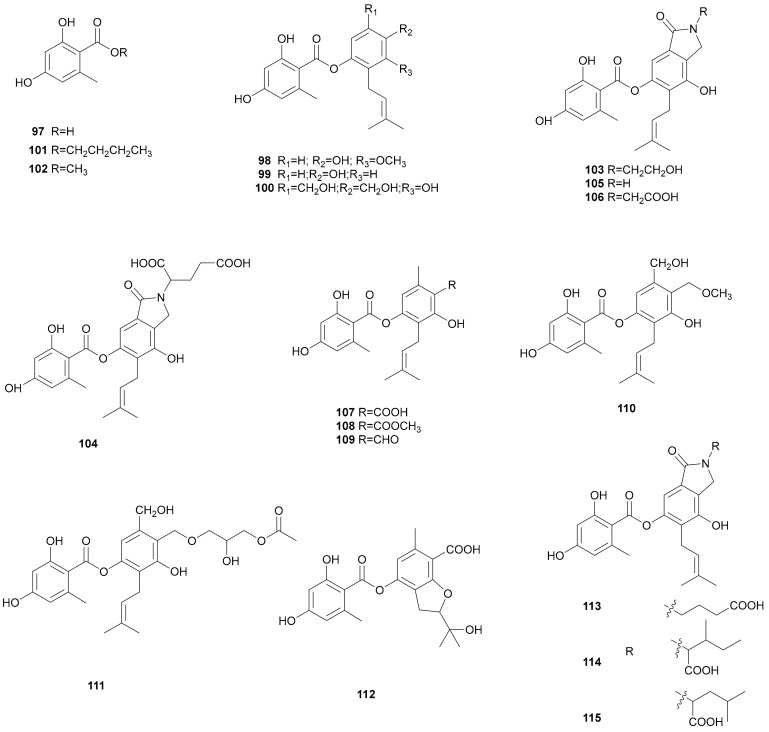
Polyketides and their derivatives from *Stereum*.

The novel isoindolinone alkaloids, known as sterenins A–D (103–106), were first found in *Stereum* sp. SANK 21205 [62]. Later, they were also obtained from *S. hirsutum* [63,64]. They exhibited potent and selective inhibitory activities against 11-hydroxysteroid dehydrogenase type 1. Sterenins A–C (103–105) displayed inhibitory activities against yeast α-glucosidase with IC_50_ values of 25.10, 12.32 and 3.31 µM, respectively [63]. Sterenin D (106) showed 67% inhibition of mycelial growth of *Botrytis cinerea* at 1000 mg mL^−1^, and 76% inhibition at 2000 mg mL^−1^. Sterenin D (106) also controlled sporogenesis effectively by inhibiting 96% of the sporulation at 500 mg mL^−1^, and assays showed that sterenin D (106) exhibited a minimal fungicidal concentration of 50 mg mL^−1^ and a MIC of 20 mg mL^−1^ [64]. The first total syntheses of sterenins A, C, and D (103, 105, 106) were described by Shinozuka et al. [65]. Nine new isoprenylated depsides, sterenins E–M (107–115) were also isolated from a solid culture of *S. hirsutum*. Sterenins K–M (113–115) showed inhibitory activities against yeast α-glucosidase with IC_50_ values of 7.62, 3.06, 6.03, 22.70, 36.64, 13.09, and 27.52 µM, respectively. According to the above results, it can be inferred that the activity is closely related to the structure. The relatively strong activity of sterenins E–G (107–109) compared to sterenins H (110) and I (111) indicated that the carbonyl group on B-ring can enhance the inhibitory activity against α-glucosidase. The fact that sterenin E (107) presented much stronger activity than sterenin J (112) confirmed that the isoprenyl group made a significant contribution to the inhibitory activity. The propane-1,2,3-triol linkage at C-8 and the formation of a furan ring at C-2 and C-3 in sterenin I (111) greatly decreased their inhibitory activity [63]. Their structures are shown in Figure 6.

### 2.3. Aromatics and Their Derivatives

#### 2.3.1. Benzofuran

**Figure 7:**
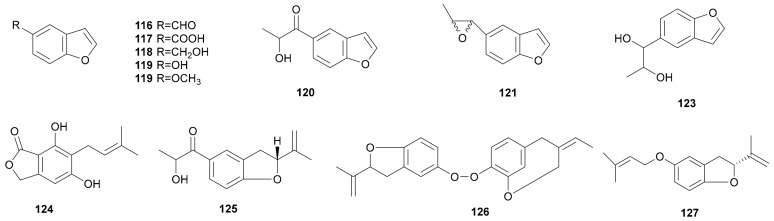
Benzofuran from *Stereum*.

Benzofuran is deemed as one of the most important heterocyclic rings on account of its diverse biological activities and clinical importance [66]. Nine benzofuran compounds 5-benzofurancarboxaldehyde (116), 5-benzofurancarboxylic acid (117), 5-benzofuranol (118), 5-methoxybenzofuran (119), 1-(5-benzofuranyl)-2-hydroxy-1-propanone (120), 5-(1,2-dihydroxy- propyl)benzofuran (121), 5-benzofuranmethanol (122) and 5-(2-methyloxiranyl)benzofuran (123) were obtained from *S. subpileatum*. 5-Methoxybenzofuran (119) had weak antibacterial activity [67]. New metabolites 5,7-dihydroxy-6-(3-methylbut-2-enyl)-isobenzofuran-1(3*H*)-one (124), phenostereum A (125), phenostereum B (126), and dihydrobenzofuran (127) were obtained from *Stereum* sp. 8954 [43], *Stereum* sp. YMF1.1684 [68] and *Stereum insigne* CGMCC5.57, respectively [69]. Yu et al. reported that phenostereum A (125) could be synthesized in an enantiopure form [70]. Their structures are shown in Figure 7.

#### 2.3.2. Phenol Derivatives and Other Aromatic Compounds

Phenol is an important part of a large variety of natural products, and presents high antimicrobial potential [71]. Many phenol derivatives have been isolated from *Stereum* species, such as 4-(2-hydroxyethyl)phenol (128) from *Stereum* sp. CCTCC AF 207024 [39]; 2-(2-hydroxyethyl)-4-methoxyphenol (129) from *S. subpileatum* [67]; 3,5-dihydroxy-4-(3-methylbut- 2-enyl)benzene-1,2-dicarbaldehyde (130) from *Stereum* sp. 8954 [61]; dibutyl phthalate (131) and (3,4-dimethoxyphenyl)methanol (132) from *Stereum* sp. YMF1.1660 [72]; 4-hydroxybenzaldehyde (133) from *S. insigne* CGMCC5.57 [69]; erinapyrone C (134) and hericenols A-D (135-138), which possess a geranyl side chain attached to a resorcinol skeleton as a common structure, and 6-hydroxymethyl-2,2-dimethylchroman-4-one (139) from *Stereum* sp. 99123 [73]; 2-(3-methyl-2- buten-1-yl)-4-methoxyethyl-phenol (140), 5-hydroxy-4-(hydroxymethyl)-2-(3-methylbut-2-en-1- yl)cyclohex-4-en-1-one (141), 3-(hydroxymethyl)-4-methoxy-2-(3-methylbut-3-en-1-yn-1-yl)phenol (142), 4-methoxy-benzoic acid methyl ester (143), and niacinamide (144) from solid fermentation extracts of *S. hirsutum* FP-91666 [74]; (3S*,4R*)-6-acetoxymethyl-2,2-dimethyl-3,4-dihydro-2H- chromene-3,4-diol (145) and (11S,12R)-vibradiol (146) from *S. vibrans* [75,76]. 3,5-Dihydroxy-4-(3- methylbut-2-enyl) benzene-1,2-dicarbaldehyde (130) showed significant nematocidal activity against the nematode *P. redivivus* [61]. Hericenols A (135) and C (137) displayed weak antimicrobial and cytotoxic activity, respectively [73]. Barrett et al. reported that hericenol A (135) could be synthesized by an efficient pathway via a regioselective palladium-catalyzed decarboxylative geranyl migration and aromatization [77]. Kobayashi et al. synthesized hericenols B–D (136–138) on the basis of a divergent assembly of a geranyl resorcylate library using an appropriately protected C5’-oxygen to combine the geranyl phthalide as a common intermediate [78].

**Figure 8:**
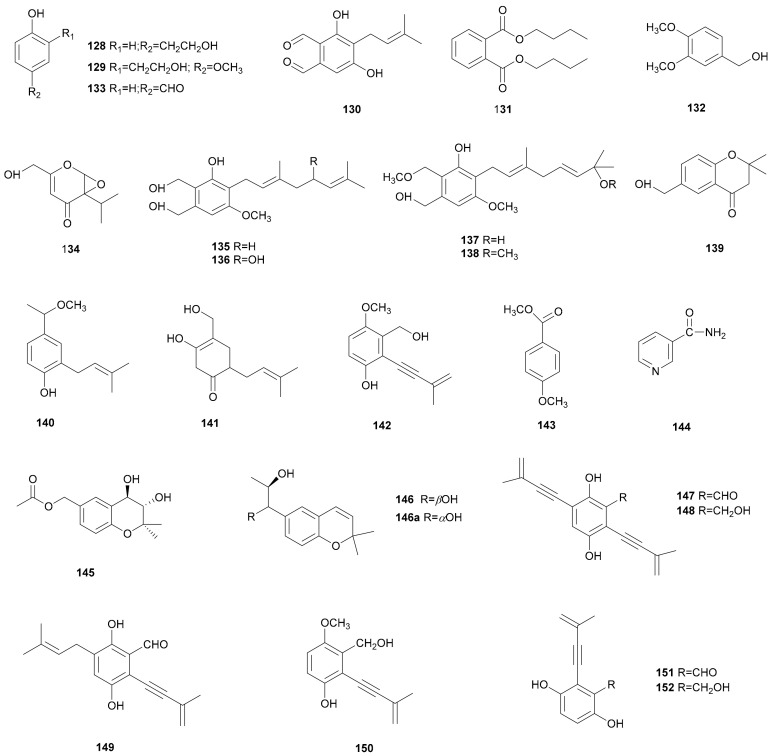
Phenol derivatives and other aromatic compounds from *Stereum*.

A series of acetylenic aromatic metabolites, including four new ones, 2,5-dihydroxy-3,6-bis(3- methylbut-3-en-1-ynyl)benzaldehyde (147), 3-(hydroxymethyl)-2,5-bis(3-methylbut-3-en-1- ynyl)benzene-1,4-diol (148), 2,5-dihydroxy-3-iso-prenyl-6-(3-methylbut-3-en-1-ynyl)benzaldehyde (149), 2-hydroxy ± 5-methoxy-6-(3-methylbut-3-en-1-ynyl) benzylalcohol (150), and the known compounds frustulosin (151) and frustulosinol (152) were obtained from *S. hirsutum* [79]. Frustulosin (151) and frustulosinol (152), which were isolated previously from cultures of *S. frustulosum*, exhibited antimicrobial activity and phytotoxicity [80,81,82]. The syntheses of frustulosin (151) and frustulosinol (152) have been realized [82,83,84]. Three different methods have been represented for the synthesis of frustulosin (151). In 1979, Alex reported that frustulosin (151) was originally assigned as a mixture of the E and Z isomers of the vinyl chloride moiety of benzofulvene [83], and that it could be converted into frustulosinol (152) [81]. Ronald et al. reported that a regioselective total synthesis of frustulosin (151) could be accomplished from 3,6-dihydroxy-2-iodobenzaldehyde [84]. Later, a new concise and efficient method to synthesize frustulosin (151) and frustulosinol (152) was developed by Goddard et al. They used a bromo analog as a key synthon to afford plenty of frustulosin with a total yield of 37% [82]. Their structures are shown in Figure 8.

### 2.4. Vibralactones and Derivatives

Vibralactone (153), a distinctive fused lactone-type compound, was isolated from *S. vibrans* (syn. *Boreostereum vibrans*) and found to inhibit pancreatic lipase with an IC_50_ of 0.4 μg mL^−1^. It is a lead compound with a novel skeleton structure and is a potential candidate for a new antiobesity therapeutic [6,7]. The absolute configuration of vibralactone (153) was assigned through optical rotation calculation. Its structure is similar to orlistat, an α-lactone-type natural lipase inhibitor, which can block the hydrolysis of triglycerides to inhibit gastric and pancreatic lipase and thus break the uptake of fatty acids from the diet [85]. The α-lactone is critical in the inhibition process, and it can covalently bind to the active serine site via acylation. The same pancreatic lipase inhibitory mechanism probably occurs on vibralactone. Recently, vibralactone (153) was used as a probe to research the activity and structure of the ClpP1P2 complex from *Listeria monocytogenes* [86]. In 2008, the total syntheses of racemic and (-)-vibralactone were reported by Snider et al. They formed the C1 all-carbon quaternary center with high diastereoselectivity through an auxiliary-controlled Birch reduction−prenylation strategy [87]. Later, Alexander reported that a total synthesis of (±)-vibralactone could be achieved in eleven steps and 16% overall yield from malonic acid [88]. Later, a series of vibralactone-related compounds, vibralactones B–Q (154–169), 1,5-secovibralactone (173), and acetylated vibralactone (174) were obtained from cultures of the *S. vibrans* [75,89,90,91,92]. The absolute configuration of vibralactone B (154) was amended by X-ray analysis [76]. The absolute configuration of 1,5-secovibralactone (173) was suggested to be S by computational methods [89]. The relative configuration of vibralactone D (156) was confirmed by X-ray analysis, while the absolute configurations of vibralactones D–F (156–158) were established by Mosher’s method [90]. Vibralactone (153) and vibralactone B (154) were also obtained from *S. rameale* and *S. hirsutum* (Sh134-11) [64]. The two compounds showed antibacterial activity against gram-negative bacteria. The MIC of vibralactone (153) against *E. coli* was 200 μg mL^−1^; vibralactone B (154) inhibited the growth of *Pseudomonas aeruginosa* and *E. coli* significantly, with MIC values of 100 and 50 μg mL^−1^, respectively [60]. Vibralactone D (156) showed inhibitory activities against 11ß-hydroxysteroid dehydrogenase type 1 (human IC_50_ 85.7 μM; mouse IC_50_ 295.2 μM) and 11ß-HSD2 (human IC_50_ 87.1 μM). Vibralactones E (157) and F (158) showed weak activity against human 11ß-HSD1 with suppression rate of 43.6% and 31.2% at 150 mg mL^−1^ and they also against type 1 mouse ß-hydroxysteroid dehydrogenase with suppression rates of 37.7% and 24.8% at the same concentration [90]. The biosynthesis of vibralactone (153) and vibralactone-associated compounds have been illustrated [93,94]. It was demonstrated that all these compounds shared the biosynthetic precursor 3-prenyl-4-hydroxybenzylalcohol. A FAD-binding monooxygenase (VibMO1) from *S. vibrans* that converts prenyl-4-hydroxybenzoate into prenylhydroquinone was also characterized. Vibralactones R (170) and S (171) were isolated from an undescribed stereaceous basidiomycete belonging to the family Stereaceae [95]. Vibralactones G–J (159–162), L (164), M (165) and R (170), sharing the γ-lactone moiety, presumably derive from 1,5-seco-vibralactone by ester hydrolysis and decarboxylation.

**Figure 9:**
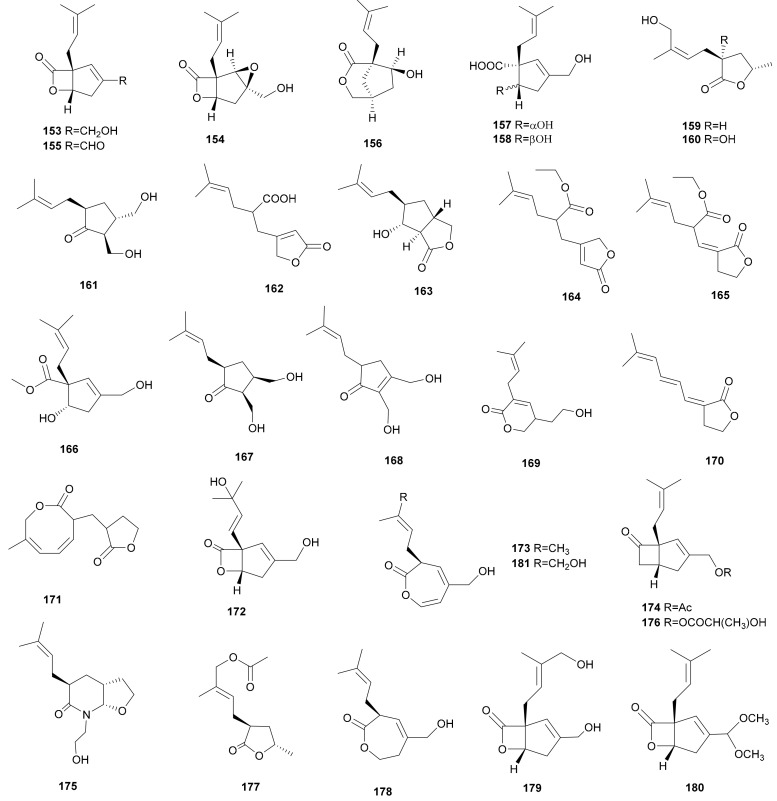
Vibralactones and derivatives from *Stereum*.

Vibralactone T (172), vibralactamide A (175), 13-O-lactyl vibralactone (176) and 10-O-acetyl vibralactone G (177) were obtained from the fungus *S. vibrans* [76]. A new vibralactone derivative, dihydro-1,5-secovibralactone (178), was isolated from *Stereum* sp. OUPS-124D-1 metabolites [96]. Ostalactones A-C (179-181), three new β- and ε-lactone compounds, were isolated from the *S. ostrea.* Ostalactones A (179) and B (180) exhibited strong inhibitory activity against human pancreatic lipase [97]. Their structures are shown in Figure 9.

### 2.5. Lanostane Triterpenoids

**Figure 10:**
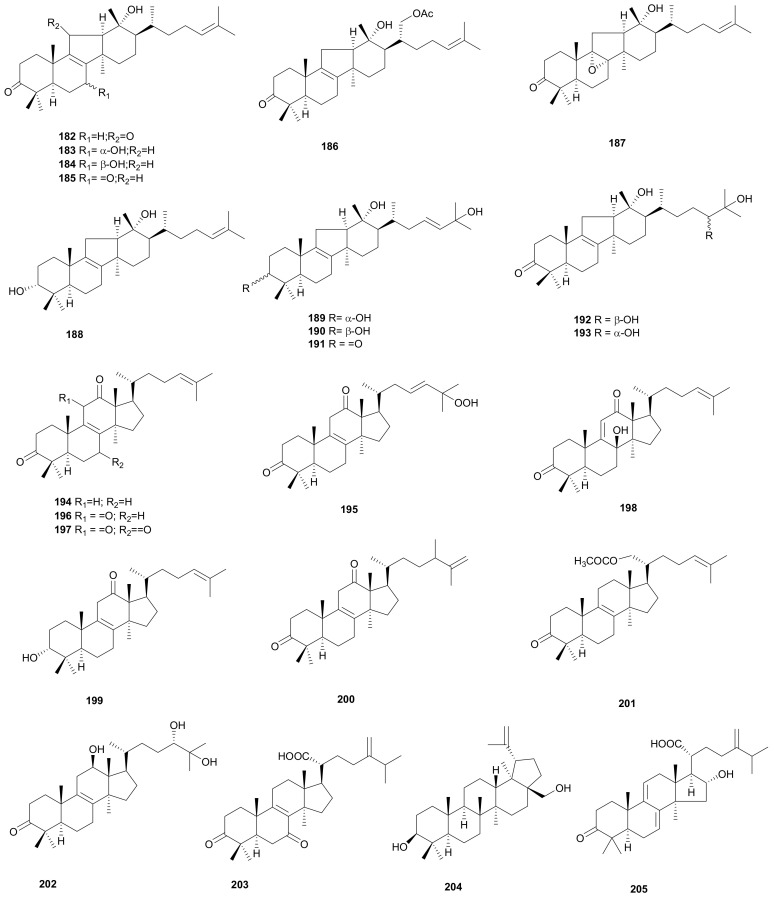
Lanostane triterpenoids from *Stereum*.

Sterenoids A–L (182–193), twelve lanostane triterpenoids, have been obtained from fruit bodies of *Stereum* sp. [98], and their absolute configurations were confirmed by unbiased quantum chemical NMR and ECD calculations. The 13R configuration in these compounds features a distinctive and rare 14(13→12) *abeo*-lanostane type [99,100]. Sterenoid E (186) exhibited effective cytotoxicity against tumor cell lines SMMC-7721 and HL-60 with IC_50_ values of 7.6 and 4.7 μM, respectively [98]. Stereinones A–J (194–203), ten highly-oxygenated lanostane triterpenoids, were obtained from the *Stereum* sp. Stereinones A–G (194–200) share the carbonyl group at C-12. Stereinones C (196) and D (197) were lanostane-type triterpenoids that contained an unusual 1,2-diketone functional group at C-11 and C-12. The absolute configuration of C-24 in stereinone I (202) was assigned by a Mo_2_(OAc)_4_-induced CD experiment for vicinal diols. Stereinone D (197) showed modest cytotoxic activity against tumor cell lines SW480 (IC_50_ 9.8 μM) and SMMC-7721 (IC_50_ 9.1 μM) [101]. Two triterpenoid compounds, betulin (204) and polyporenic acid C (205) were obtained from the fruit body of *S. subtomentosum* [102]. Their structures are shown in Figure 10. 

### 2.6. Sterols

Sterols have long been regarded as important compounds in drug discovery because of their varied biological activities, such as cytotoxicity, neurite outgrowth-promoting activity, anti-NO production and acetylcholinesterase inhibition [103]. Steroids are predominant secondary metabolites with various structural features, both their isolation and synthesis have gained attention in recent years [104,105].

Most of the sterols from *Stereum* were obtained from *S. hirsutum*, including epidioxysterols 1-4 (206-209), ergosterol peroxide (210), (22*E*,24R)-ergosta-5,7,22-trien-3*β*-ol (211), 3*β*,5α,6*β*-triol-ergosta- 7,22-diene (212), 3*β*,5α-diol-6*β*-methoxy-ergosta-7,22-diene (213), 3*β*,5α,9α-trihydroxy-ergosta- 7,22-dien-6-one (214), steresterones A (215), B (216), 3β,6α,14α-trihydroxy-dankasterone A (217a), 3α,6α,14α-trihydroxy-dankasterone A (217b), dankasterones A (217) and B (218), herbarulide (219), (14α,22E)-14-hydroxy-ergosta-4,7,22-triene-3,6-dione (220), ergosta-4,7,22-triene-3,6-dione (221), isocyathisterol (222), (14β,22*E*)-9,14-dihydroxyergosta-4,7,22-triene-3,6-dione (223), and (22E)-ergosta-4,22-diene-3,6-dione (224) [74,106,107]. The absolute configuration of steresterone B (216) was determined by the comparison of experimental and theoretical ECD. The structure of dankasterone A (217), which contained a 5β-H was confirmed by X-ray diffraction analysis [106]. Epidioxysterols 1 (206) and 2 (207) displayed activity against *M. tuberculosis* H37Rv reference strain with an MIC of 16 μg mL^−1^, while epidioxysterols 3 (208) and 4 (209) showed the same MIC of 64 μg mL^−1^ [107]. Ergosterol peroxide (210) was isolated from *S. subtomentosum* [102] and *S. insigne* CGMCC5.57 [69]. It has demonstrated many available biological properties, such as antimycobacterial [108], antiplasmodial [109], immunosuppressive [110], antiviral, anti-inflammatory, antitumor activities [111], and enhancement of the suppression effect of linoleic acid on DNA polymerase b [112]. Recently, Ling et al. presented an efficient synthesis of ergosterol peroxide (210), chemical probe for in live-cell studies, vitro anticancer evaluation and proteomic profiling. Dankasterones A (217) and B (218) displayed effective inhibition against the murine P388 cancer cell line. Compounds dankasterones A (217) and B (218), (14α,22E)-14-hydroxyergosta-4,7,22-triene-3,6-dione (219), (14β,22E)-9,14-dihydroxyergosta-4,7,22-triene-3,6-dione (220) and isocyathisterol (222) showed cytotoxicity against five cell lines: HL-60, A549, SW480, MCF and SMMC-7721, with IC_50_ values ranging from 2.3 to 25.7 μM. Ergosta-4,7,22-triene-3,6-dione (221) showed only weak cytotoxicity against the HL-60 cell line with an IC_50_ of 34.3 μM. Dankasterone B (218) showed strong cytotoxicity against five human cancer cell lines (HL-60, SMMC-7721, A549, SW-480, and MCF-7), with IC_50_ values of 2.3, 3.3, 4.4, 3.5, and 2.7 μM, respectively. (14α,22E)-9,14-Hydroxyergosta-4,7,22-triene-3,6-dione (220) also displayed significant cytotoxic activity against the same five cell lines with IC_50_ values of 3.1, 9.0, 11.0, 13.2 and 12.3 μM, respectively. Intriguingly, although the compounds dankasterones A (217) and B (218) share similar structures, with the exception of substituents between C-4–C-5, dankasterone B (218) showed stronger cytotoxicity than dankasterone A (217). This result suggests that the presence of C-4–C-5 double bond plays a significant role in mediating the cytotoxic activity against the cancer cell lines tested. Steresterone B (216), containing a hydroperoxyl moiety at C-14, showed no cytotoxicity. However, (14α,22E)-14-hydroxyergosta-4,7,22-triene-3,6-dione (220), an analog of steresterone B (216) with a hydroxy at C-14, showed greater cytotoxic activities in these tests. The data revealed that the functional group 14-OH is a key assistant for its cytotoxic effects. Two compounds 3β, 6α, 14α-trihydroxydankasterone A (217a) and 3α,6α,14α-trihydroxydankasterone A (217b) were semi-synthesized from dankasterone A (217) because of its relatively high yield and moderate cytotoxicity, and neither of them showed improved cytotoxicity. The above results indicated that the carbonyl groups on dankasterone A (217) were essential for its cytotoxicity.

**Figure 11:**
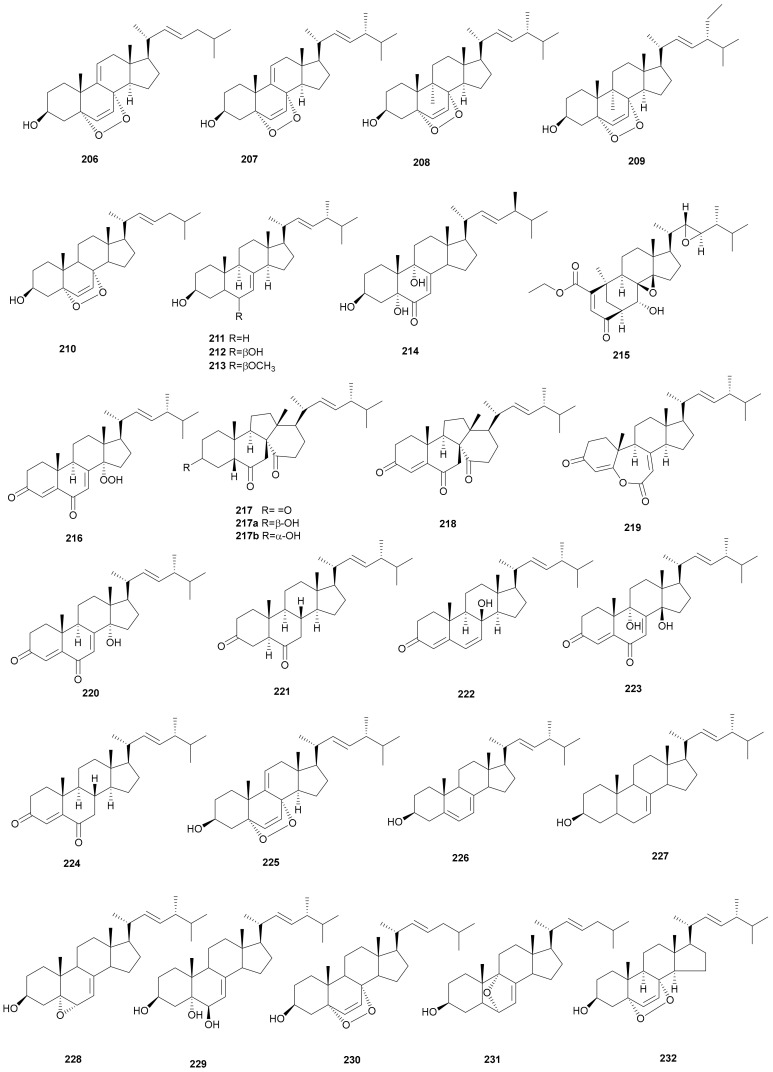
Sterols from *Stereum*.

Apart from *S. hirsutum*, sterols have also been isolated from other species of *Stereum*. For example, dehydroergosterol peroxide (225), and (22*E*,24R)-ergosta-7,22-dien-3*β*-ol (226), were obtained from the fruit body of *S. subtomentosum* [102]. Stella sterol (227), (3β, 5α, 6α, 22*E*)-3-hydroxy-5,6-epoxy-7-one-8(14),22-dien-ergosta (228), and (3β,5α,6β,22E)- 7,22-diene-3,5,6-triol-ergosta (229) were obtained from the mycelium of *S. insigne* CGMCC5.57 [69]. 5-Epidioxyergosta-6,22-dien-3-ol (230) and 6,9-epoxy-ergosta-7,22-dien-3-ol (231) were isolated from *Stereum* sp. YMF1.1660 [72], and (3β,5α,22E,24R)-5,8-epidioxyergosta-6,22-dien-3-ol (232) was obtained from the fungus *Stereum* sp. CCTCC AF 207024 [39]. Dehydroergosterol peroxide (225) displayed cytotoxicity against HT29 colon adenocarcinoma cell and HL60 leukemia [113]. Their structures are shown in Figure 11.

### 2.7. Carboxylic Acids and Saccharides

**Figure 12:**
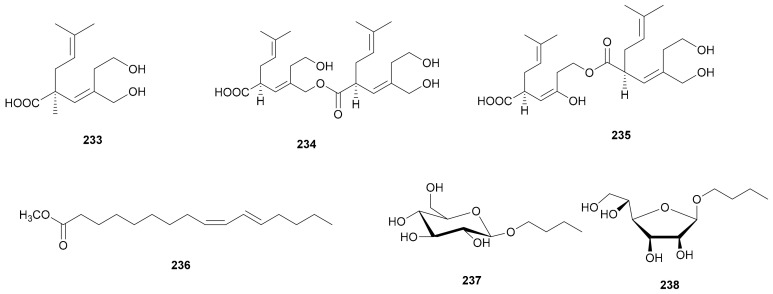
Carboxylic acids and saccharides from *Stereum*.

Three carboxylic acids with two primary alcohols, sterepinic acids A–C (233–235), have been isolated from *Stereum* sp. OUPS-124D-1 and their absolute configurations were determined by the application of the phenylglycine methyl ester (PGME) method [96]. Methyl ester-(9*Z*,11*E*)-9,11-hexadecadienoic acid (236) was obtained from *S. insigne* CGMCC5.57 [69]. In addition, two saccharides butyl-β-D-glucopyranoside (237) and butyl-β-D-glucofuranoside (238) (Figure 12) were isolated from *Stereum* sp. YMF1.1660 [72]. Their structures are shown in Figure 12.

## 3. Conclusions

Over the past 70 years, 238 compounds have been discovered from the genus of *Stereum*, nearly half of which were sesquiterpenoids, and some of them were only found in *Stereum*. The results have shown that the production of secondary metabolites by organisms is not random, but related to their ecological niches [114]. We speculate that the production of sesquiterpenoids may be a characteristic of *Stereum*, especially since the bioactive ones may help *Stereum* to defend their habitat or to suppress the growth of competitors to maintain its own abundance.

According to the studies, we found that some bioactivities of metabolites may be related to critical bonds or groups of structure. For example, sterhirsutins E (15) and G (17) (Figure 1), the immunosuppressant bioactivity depends on the ether bond between C-15′ and C-16′. When sterenins E–G (107–109) are compared with sterenins H (110) and I (111) (Figure 6), the strength of inhibitory activity against α-glucosidase is related to the carbonyl and isoprenyl groups. When we compare dankasterone A (217) with dankasterone B (218) (Figure 11), it seems that the carbonyl groups in the structure are essential for its cytotoxicity. Therefore, we may get more bioactive compounds by changing specific bonds and groups through chemical processes.

The discovery of novel and bioactive secondary metabolites is a target of drug and natural product chemists. Microorganisms are dominant sources for such substances. The metabolites of *Stereum* have been a potential drug repository for antiobesity therapeutics, cancer, inflammation, and other diseases, but it is difficult to obtain large quantities of active compounds because only minor amounts can be obtained from natural sources. As a result, we should do more research into the synthesis of such compounds. Recently, SteTC1, a type I cembrane diterpene synthetase from *S. histurum*, was characterized via genomic data analysis, phylogenetic method, protein sequence alignment and products detection with GC-MS [115]. This is instructive for scientists to do research using integrated approaches. There are 27 species in *Stereum*, but only 10 known species have been studied for their metabolites, so more new and active compounds are likely to be discovered from *Stereum* in the future.
